# Short-term ocean acidification decreases pulsation and growth of the widespread soft coral *Xenia umbellata*

**DOI:** 10.1371/journal.pone.0294470

**Published:** 2023-11-15

**Authors:** Arjen Tilstra, Lorena Braxator, Bianca Thobor, Selma D. Mezger, Claudia E. L. Hill, Yusuf C. El-Khaled, Giulia Caporale, Sohyoung Kim, Christian Wild

**Affiliations:** Department of Marine Ecology, University of Bremen, Bremen, Germany; ULAB: University of Liberal Arts Bangladesh, BANGLADESH

## Abstract

Coral reefs may experience lower pH values as a result of ocean acidification (OA), which has negative consequences, particularly for calcifying organisms. Thus far, the effects of this global factor have been mainly investigated on hard corals, while the effects on soft corals remain relatively understudied. We therefore carried out a manipulative aquarium experiment for 21 days to study the response of the widespread pulsating soft coral *Xenia umbellata* to simulated OA conditions. We gradually decreased the pH from ambient (~8.3) to three consecutive 7-day long pH treatments of 8.0, 7.8, and 7.6, using a CO_2_ dosing system. Monitored response variables included pulsation rate, specific growth rate, visual coloration, survival, Symbiodiniaceae cell densities and chlorophyll *a* content, photosynthesis and respiration, and finally stable isotopes of carbon (C) and nitrogen (N) as well as CN content. Pulsation decreased compared to controls with each consecutive lowering of the pH, i.e., 17% at pH 8.0, 26% at pH 7.8 and 32% at pH 7.6, accompanied by an initial decrease in growth rates of ~60% at pH 8.0, not decreasing further at lower pH. An 8.3 ‰ decrease of δ^13^C confirmed that OA exposed colonies had a higher uptake and availability of atmospheric CO_2_. Coral productivity, i.e., photosynthesis, was not affected by higher dissolved inorganic C availability and none of the remaining response variables showed any significant differences. Our findings suggest that pulsation is a phenotypically plastic mechanism for *X*. *umbellata* to adjust to different pH values, resulting in reduced growth rates only, while maintaining high productivity. Consequently, pulsation may allow *X*. *umbellata* to inhabit a broad pH range with minimal effects on its overall health. This resilience may contribute to the competitive advantage that soft corals, particularly *X*. *umbellata*, have over hard corals.

## Introduction

Coral reefs are under threat from a variety of factors, including anthropogenically induced ocean acidification (OA) [1]. Ocean acidification is characterized by a drop in pH caused by the increased dissolution of atmospheric CO_2_ in ocean water [2]. Since the start of industrialization and the subsequent increase of atmospheric CO_2_ concentrations, the pH of the world’s oceans has already decreased by 0.1 units and is currently at an average of 8.1 [3, 4]. This value is expected to drop further by 0.3 to 0.4 units over the next 100 years [5, 6] if current CO_2_ emissions persist. Simultaneously, because of the more acidic water, the aragonite saturation state decreases [7], rendering calcifying organisms especially vulnerable [8].

Scleractinian, or hard corals, i.e., the ecosystem engineers of coral reefs [9], are an example of such. The effects of OA on hard corals are usually negative and can be direct, e.g., by reduced calcification rates [10, 11], reduced sexual recruitment [12], reduced fixation of essential nitrogen by diazotrophs [13], and increased macrobioerosion [14], or indirect, e.g., from coral competition and (macro)algal interactions [15]. Some studies report high interspecific variability [16] and severity [17] in hard corals’ responses, while others report short-term resistance [18], or even positive effects by benefiting photophysiological measures [19].

The focus of OA research has been primarily on hard corals while the second biggest taxon on coral reefs, i.e., soft corals, especially from tropical regions, are relatively overlooked. Soft corals may overtake reefs as the dominant taxon after die-offs of hard coral [20–23]. Despite the lower structural complexity that comes with soft coral dominance (compared to hard coral dominance), they may still provide important habitat to e.g., reef fishes [24, 25]. Some OA studies on tropical soft corals reported no negative effects on the corals’ physiology [26, 27], while others reported relatively minor negative effects [28, 29]. Gabay and colleagues [26, 27] suggested that the tissue of soft corals may act as a protective barrier against OA associated physiological and morphological change, i.e., the dissolution of calcium carbonate sclerites in their hydroskeleton. Ultimately, this may differentiate soft corals from hard corals in their response to OA.

Soft corals of the Xeniidae family are particularly successful, both as native spreaders and non-native invaders [30, 31]. Because of their extensive vegetative reproduction with high growth rates, recruitment abilities, high fecundity, and extended annual planulation, these colony-forming soft corals often take over disturbed habitats [32–34]. Like most hard corals, xeniids are photosymbiotic animals living in close association with endosymbiotic dinoflagellates of the family Symbiodiniaceae [35], which enables effective utilization and storage of nutrients and photosynthates (i.e., photosynthetically fixed carbon). Furthermore, the characteristic pulsating movement of some xeniid species effectively prevents refiltration by neighboring polyps through the induced upward movement of water [36], thereby increasing photosynthesis, heterotrophic feeding, and nutrient uptake [37]. The pulsation of xeniids is not always consistent, however, and can change according to the environmental conditions the xeniid is exposed to [38–41]. Pulsation may thus be used as a first indicator for environmental change.

The current study aimed to assess the effects of short-term OA on the physiology of the pulsating xeniid species *Xenia umbellata*. To do so, we investigated the ecological response of the coral holobiont based on pulsation rate, specific growth rate (SGR), visual coloration, survival, Symbiodiniaceae cell densities, chlorophyll *a* (chl. *a*) content, oxygen fluxes, carbon (C) and nitrogen (N) isotope signatures, and CN content. We hypothesized, based on previous research, that OA would increase the incorporation of lighter C isotopes due to higher atmospherically derived dissolved inorganic C (DIC) availability [42], but that further response variables would remain unaffected [26]. However, in case C was the limiting factor for primary production, we hypothesized an increase in net photosynthesis and respiration [43], followed by increased pulsation rates and holobiont C and N content.

## Materials and methods

### Sample species, setup, and maintenance

Fragments of *X*. *umbellata* were taken from several mother colonies of the same genotype that have been cultured under stable conditions for several years in the aquarium facilities at the University of Bremen Marine Ecology department. This particular genotype was purchased at a retail shop in Germany and originally sampled in the Red Sea. Colonies from the main holding aquarium were fragmented following the plug mesh method by Kim and colleagues [44]. In brief, colonies were cut into smaller 2 cm pieces and secured to a calcium carbonate plug (AF Plug Rocks, Aquaforest, Poland), creating a total of 132 fragments. Fragments were randomly distributed over 12 independent glass aquaria (60 L) on 40 x 20 cm plastic grids, with a minimum of 2 cm between each fragment, resulting in 11 fragments per aquarium.

Each aquarium was divided into 1) a technical part containing a heating element (EHEIM thermo control, 50W, EHEIM, Germany, accuracy ± 0.5°C), which was sufficient to keep the water temperatures stable, a return pump for water circulation (EHEIM CompactOn 300 pump, EHEIM, Germany), and a pendant logger (HOBO pendant, Onset, USA, accuracy ± 0.5°C) for constant measurements of temperature and light, and 2) an experimental part housing the corals. Both parts were separated by a glass wall with an overflow but had a consistent water exchange using the previously described return pump. The light was provided by LED lamps (Royal blue matrix module and ultra-white blue 1:3-matrix module WALTRON daytime) in a 12:12 h day-night cycle at a PAR intensity of ~100 μmol photons m^−2^ s^−1^. Tanks were filled with unfiltered artificial seawater, which was created by adding aquarium sea salt (Zoo Mix, Tropic Marin, Switzerland) in a barrel with demineralized water containing a heating element and circulation pump. Salinity and temperature were checked daily using a portable multimeter (HACH HQ40D portable multimeter, United States, accuracy ± 0.5). For salinity, a value of 35 ‰ was targeted, while temperature was kept at 25.7 ± 0.3°C. Nitrate, nitrite, ammonium and phosphate were measured twice per week, calcium and magnesium were measured once per week, and alkalinity was tested daily using JBL TestLab Marin test kits. Water parameters (except for pH) of all tanks were constantly maintained throughout the entire experiment (see Table 1). Biofouling on glass surfaces was removed regularly without physically disturbing the fragments.

**Table 1:** Mean ± S.D. (if applicable) of water/environmental parameters maintained in all tanks.

Parameter	Mean values (± S.D.)
Temperature	25.7 ± 0.3°C
Salinity	35.1 ± 0.1 ‰
PAR	~100 μmol m^−2^ s^−1^
Nitrate	< 0.5 ppm
Nitrite	< 0.01 ppm
Ammonium	< 0.05 ppm
Phosphate	< 0.02 ppm
Calcium	377 ± 25 ppm
Magnesium	1327 ± 72 ppm
Alkalinity	8 ± 2 dKH

PAR = Photosynthetically Active Radiation

### Experimental design

The experiment was run for 21 days with each of the three treatments lasting 7 days as previous research conducted with the same organism resulted in reactions of response variables within this timeframe [38, 39]. Controls and OA treatments were each replicated in six aquaria (n_treatment_ = 6), randomly arranged in a three-level tower with four tanks per level to ensure equal representation.

The acidification of the water took place in three stages by sequentially decreasing pH-levels from ambient, i.e., the pH of our holding tank: pH of ~8.3 ± 0.1, to 8.0, 7.8, and finally to 7.6, each of which was maintained for a full week. According to the IPCC, pH 8.0 and 7.8 represent values that will be reached within the next decades under the RCP8.5 scenario [6], while a pH of 7.6 is an even more extreme value than expected by IPCC scenarios.

The water within six aquaria was acidified using a CO_2_ system (S1 Fig) while maintaining stable alkalinity. A pH computer (NBS; pH computer set, Aqua Medic, accuracy 0.01 pH) was used to keep the pH stable. A CO_2_ reactor (Aqua Medic) was used to dissolve CO_2_ bubbles in the water. This reactor was connected via 4/6 mm tubing with fine needle valves and check valves to prevent backflow of water, a solenoid valve (M-valve Standard, Aqua Medic) for control, a CO_2_ cylinder (Dupla), and a pressure reducer (Aqua Medic) [45].

### Ecological assessments

To compare between treatments, pulsation, growth, coloration, survival, and oxygen fluxes were measured after each one-week period at a certain pH level, thus three times in total. Chlorophyll *a*, isotope signatures and CN content were only measured at the end of the experiment on day 21.

### Pulsation rates

Polyp pulsation was counted for 30 seconds, and one pulsation was defined as the motion of a polyp from being fully closed to opened to closed again [39]. The results were extrapolated to one minute to allow for comparisons with previous studies. For each tank, the pulsation of one polyp from three separate fragments, i.e., 36 fragments in total, the same fragments every week, was counted and averaged for further analysis. These three pseudo-replicates were averaged for statistical analyses, resulting in six tank replicates per treatment. The circulation pump of each respective tank was turned off 1 minute before the start of counting. Counting started approximately 10 minutes after the start of the light cycle in the morning to avoid differences due to circadian rhythms.

#### Specific growth rate

The estimate the SGR, all polyps of marked fragments were counted manually using tweezers while being submerged at all times to reduce further stress. Three colonies per aquarium were considered for SGR. These three pseudo-replicates were averaged for statistical analyses, resulting in six tank replicates per treatment. The SGR was calculated using the following equation [46, 47]:

$$SGR\left(d^{-1}\right)=\frac{\text{ln}\;P_{t}-\text{ln}\;P_{t-1}}{\Delta t}$$ (1)

*P*_t_ and *P*_t-1_ describe the final and the initial number of polyps, respectively, while Δt is the growth interval in days. The final growth rate unit is polyp polyp^-1^ d^-1^ which can be simplified to d^-1^.

#### Visual coloration

A total of 12 colonies (one per tank) were examined weekly for visual coloration as an indicator of bleaching according to Thobor and colleagues [38]. Briefly, photos were taken weekly with an Olympus TG6 underwater camera, with fixed manual settings (ISO 100, f/1.4, x4 magnification), and under identical light conditions. For correcting the white balance and obtaining red, green, and blue (RGB) pixel values, Adobe Photoshop 2020 was used. Color values from the tentacles of five randomly chosen polyps (25 x 25-pixel square) were averaged per colony. The RGB values were then averaged per treatment per day and the resulting #HEX color was reported visually. The use of one fragment per tank was representative of all fragments in their respective tanks (Tilstra, personal observation).

#### Survival

All colonies were monitored for survival throughout the experiment. Due to the high regeneration capacity of *X*. *umbellata* [48], colonies were only considered dead when they completely disappeared from the plug.

#### Symbiodiniaceae cell density and chlorophyll *a* content

On every measurement day, 12 colonies (one colony per tank) were randomly chosen and frozen at -20°C until further processing. Upon processing, samples were thawed, 10 mL of demineralized water was added, and homogenized (MONIPA^™^ High Speed Homogenizer FSH-2A) into a slurry. To separate the coral tissue and the Symbiodiniaceae cells, the slurry was centrifuged for 10 mins at 6000 rpm. The supernatant was discarded, and the remaining pellet was resuspended in 2 mL distilled water and again centrifuged at 6000 rpm for another 10 mins in order to further separate the coral tissue and the Symbiodiniaceae cells. The supernatant was again discarded and the remaining pellet was resuspended in 2 mL of distilled water. To count the Symbiodiniaceae cells, 10 μL of resuspended cells were loaded on both grids of a counting chamber (Neubauer^™^ counting chamber, 0.1 mm depth). Cells were then counted using a microscope (DN-107T Digital Microscope, Xiamen Phio Scientific Instruments Co., Ltd). Cell counts from both grids were averaged for downstream analysis. Symbiodiniaceae cells were normalized to the surface area to obtain the cell density (Symbiodiniaceae cells cm^-2^).

Chlorophyll *a* was measured according to Jeffrey and Humprey [49] at the end of the experiment (day 21). Briefly, a pellet with known Symbiodiniaceae cell count was resuspended in 90% acetone, vortexed and left in darkness for 24 h at 4 °C. After centrifugation, the supernatant was transferred to two 1 mL glass cuvettes. Chlorophyll *a* content was then measured in total darkness using a Trilogy Fluorometer (Turner Designs) fitted with a chl. *a* module against a pre-made calibration curve. Each sample was measured three times resulting in two times three measurements per treatment sample. Replicates were averaged and normalized per Symbiodiniaceae cell.

#### Oxygen fluxes

Net photosynthesis (*P*_net_) and dark respiration (*R*_dark_) rates were assessed by oxygen flow with light and dark incubations [50, 51].

The same colonies were used as for coloration in order to establish a potential connection.

Briefly, the respective colony (one colony per tank) was placed in a 160 mL glass jar containing water from its respective tank. The jar was sealed airtight avoiding capture of air bubbles, and placed in a water bath with a constant temperature of 26°C and ~100 μmol photons m^−2^ s^−1^ of light for *P*_net_ measurements using the same LED lights as the experimental tanks. Constant water mixing in the jars was ensured by using stirring plates with 190 rpm (Poly 15, Thermo Scientific VARIOMAG^®^ Magnetic Stirrers) and a magnetic stirrer in each jar. The oxygen concentration was measured at the start, i.e., before closing the lid, as well as at the end of the incubation, i.e., after 1 h in the light for *P*_net_ and 1 h in total darkness for *R*_dark_, using an optode sensor (Hach IntelliCAL/Optical Dissolved Oxygen Probe). Dark respiration is presented as a negative value. Gross photosynthesis (*P*_gross_) was calculated using the following equation:

$$P_{\text{gross}}=P_{\text{net}}-\;R_{\text{dark}}$$ (2)

Oxygen measurements were normalized to the surface area to obtain oxygen fluxes (μg O_2_ cm^-2^ h^-1^).

#### Coral colony surface area

Surface area for each respective individual colony was obtained to normalize Symbiodiniaceae cell density and oxygen fluxes. The surface area of the polyps (1), their stems (2) and the main colony stem (3) together produced the total colony surface area, similar to Bednarz and colleagues [52].

(1) For each colony, several photos were taken of fully protruded polyps, perpendicular to the camera to avoid errors arising from different angles. Then, colonies were taken from their respective tank and polyps were manually counted using tweezers, always by the same person at the same time to minimize observer bias and size differences due to circadian rhythms, respectively. The counted polyps were divided into two size classes, i.e., large (>6 mm) and small (<6 mm). Photos were then evaluated with ImageJ using the Freehand Tool, measuring the surface area of six polyps (three large polyps and three small polyps) each. The mean surface area of each size class was then multiplied by the number of polyps in each size class. (2) The length and diameter of five polyp stems were measured and averaged. The surface area was calculated as a cylinder (Eq 3) and multiplied by the total number of polyps of the colony. (3) Finally, the length (*h*) and diameter (2*r*) of the main colony stem was measured, and surface area (*SA*) was obtained by using the following equation:

SA=2πrh (3)

#### Stable isotope signatures and CN content

To assess the effects of increased DIC on the isotope signatures and elemental composition of holobiont C and N, six samples from both treatments, i.e., one colony per tank, were taken at the end of the experiment (day 21) and prepared according to Mezger and colleagues [53]. Briefly, colonies were carefully detached from the plug and thoroughly rinsed with distilled water to eliminate any traces of salt. Subsequently, the colony was placed in a plastic bag and preserved by freezing it at a temperature of -20°C. For subsequent processing, *X*. *umbellata* colonies were dried in sterile glass petri dishes at a temperature of 40°C, for a minimum of 48 h, and beyond if required, until they reached a consistent weight. Following this, the dried colonies were ground into a fine powder using a mortar and pestle. The resulting tissue powder was weighed, and 1–2 mg of the powder was then transferred into 5x9 mm tin cups (IVA Analysentechnik GmbH & Co. KG, Germany). Prepared samples were shipped to the Natural History Museum in Berlin and analyzed according to Karcher and colleagues [54]. Under increased *p*CO2, photosynthesis performed by the Symbiodiniaceae should primarily use the lighter seawater-dissolved CO_2_ instead of the heavier calcification derived HCO_3_^-^ as its C source, thus resulting in more negative δ^13^C values.

### Statistical analysis

Statistical analyses were carried out using Sigmaplot v12.0 (Systat software). All data were normally distributed (Shapiro-Wilk normality test) with homogeneity of variances (Levene’s test). Water parameters as well as pulsation rate, visual coloration, SGR, and oxygen flux data were analyzed via two-way repeated measures analysis of variance (2-way RM ANOVA) as data were obtained every week from the same colony. For this analysis, ‘Day’ and ‘Treatment’ were set as fixed factors, while tank number was used as subject. Symbiodiniaceae cell density data, which was collected from a different colony every week, was analyzed with a two-way analysis of variance (2-way ANOVA). For this analysis, ‘Day’ and ‘Treatment’ were set as fixed factors. Chlorophyll *a* content, isotope signatures, and CN content were analyzed via t-tests. Furthermore, all pairwise multiple comparison procedures were conducted to confirm the significant differences by carrying out Tukey’s post hoc multiple comparison tests. Figures were generated with R (version 2023.03.0+386) and SigmaPlot v12.0 (Systat software). All data are presented as mean ± S.E. unless stated otherwise.

## Results

### Water parameters

Water parameters, except for pH, in all aquaria remained constant throughout the experiment (Table 1). There were no significant differences between aquaria for either parameter.

The pH in the aquaria without acidification averaged 8.3 ± 0.1. The pH of six OA tanks averaged at 8.0 ± 0.1 in the first week, 7.8 ± 0.1 in the week after, and 7.6 ± 0.1 in the last week.

### Pulsation rate

**Fig 1:**
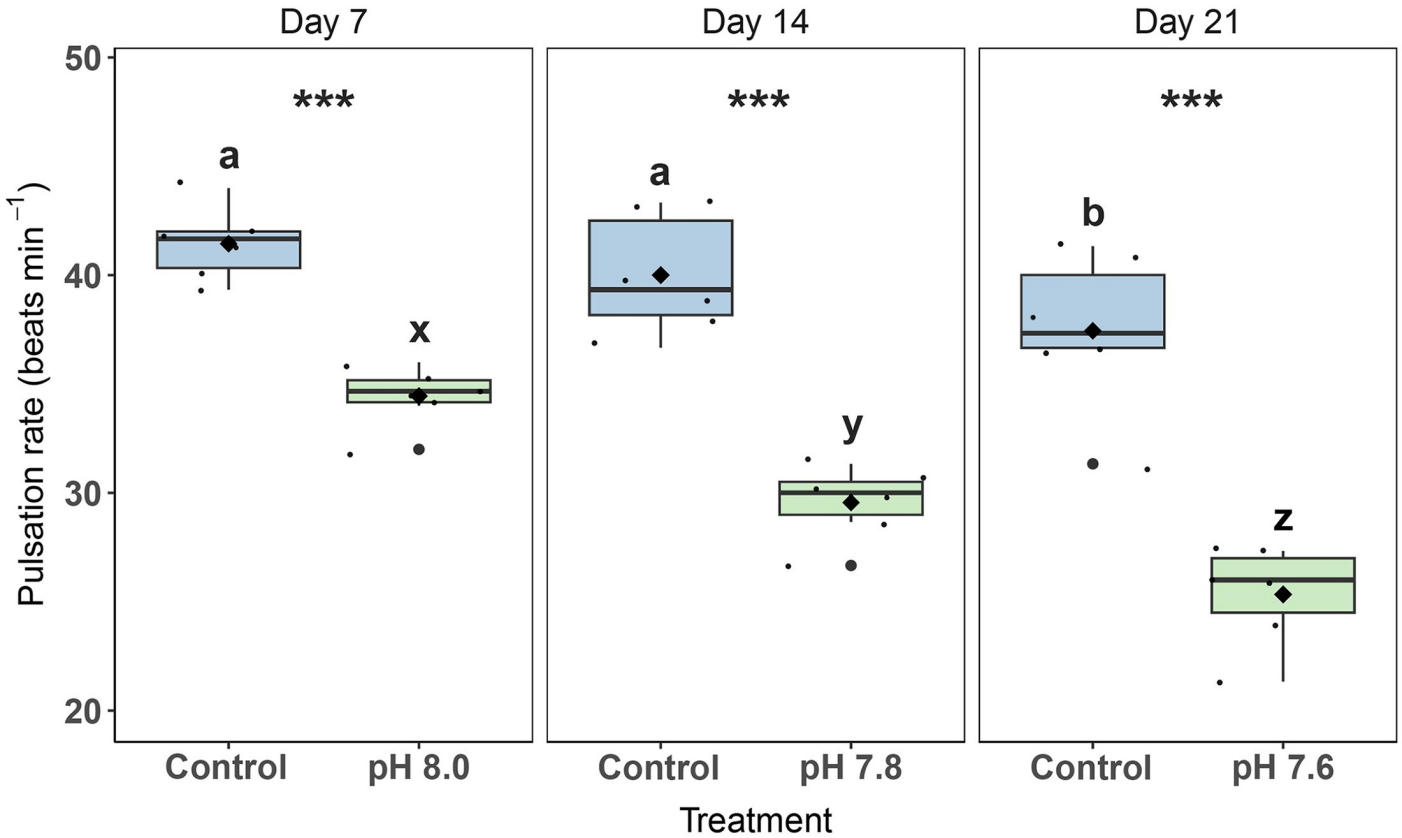
Pulsation rates of *Xenia umbellata* exposed to ocean acidification (OA). The black horizontal line in each boxplot represents the median, while the black diamond represents the mean. Blue boxplots are controls; green boxplots are the OA treatments. Small black circles represent data points (n_treatment_ = 6), and big black circles represent outliers. Significant differences (p < 0.05) within treatments between days are shown by different letters, while differences between treatments per day are shown by asterisks (*** p < 0.001).

In general, pulsation rates were lower in OA treatments compared to the control (Fig 1). There was a significant interactive effect of Day and Treatment (2-way RM ANOVA, F_2,20_ = 12.6, p < 0.001). Pulsation rates of control fragments remained constant for the first two weeks (41 ± 1 and 40 ± 1 beats min^-1^, respectively) and then decreased significantly to an average of 37 ± 1 beats min^-1^ in the final week (Fig 1). For OA treatments, pulsation rates averaged at 34 ± 1 beats min^-1^ at pH 8.0, 30 ± 1 beats min^-1^ at pH 7.8, and 25 ± 1 beats min^-1^ at pH 7.6. All OA treatments differed significantly from the controls and each other (pairwise comparison, p < 0.001) (Fig 1).

### Specific growth rate

**Fig 2:**
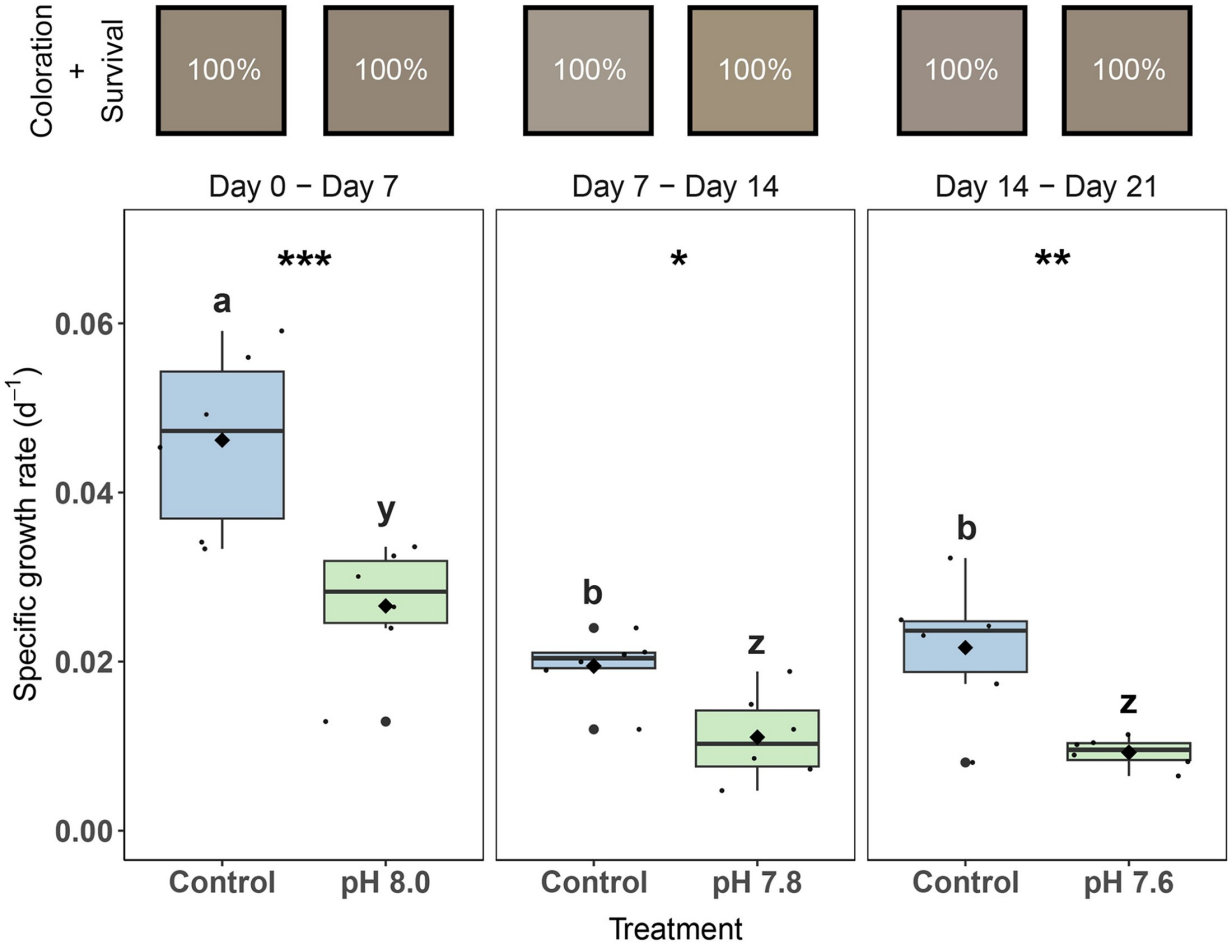
Specific growth rates, visual coloration, and survival of *Xenia umbellata* exposed to ocean acidification (OA). Color squares represent the average color of the colonies in its respective treatment (#HEX, based on Red, Green and Blue [RGB] values of photographs). Percentages inside the color squares refer to coral colonies that survived the treatment; i.e., all colonies survived in every treatment. The black horizontal line in each boxplot represents the median, while the black diamond represents the mean. Blue boxplots are controls; green boxplots are the OA treatments. Small black circles represent data points (n_treatment_ = 6), and big black circles represent outliers. Significant differences (p < 0.05) within treatments between days are shown by different letters, while differences between treatments per day are shown by asterisks (* p < 0.05, ** p < 0.01, *** p < 0.001).

Colonies exposed to OA had significantly lower SGR compared to the control during all three weeks of the experiment (pairwise comparison, p < 0.01 for all significant comparisons). Significant main effects were found for Treatment (2-way RM ANOVA, F_1,20_ = 67.6, p < 0.001) and Day (2-way RM ANOVA, F_2,20_ = 29.5, p < 0.001). The SGR for both the control and OA treatments decreased significantly (pairwise comparison, p < 0.01 for all significant comparisons) after the first week and remained stable over the last two weeks (Fig 2). Specific growth rates of control colonies decreased with 58% at day 7 –day 14 and 54% at day 14 –day 21 compared to day 0 –day 7, while SGR of OA exposed colonies with decreased with 58% and 65%, respectively (Fig 2).

### Visual coloration and survival

There were no significant differences between treatments for red, green, or blue coloration (p = 0.656, p = 0.405, p = 0.218, respectively). Overall, colors remained relatively consistent throughout the experiment and all coral fragments (100%) survived the experiment (Fig 2).

### Symbiodiniaceae cell density and chlorophyll *a* content

**Fig 3:**
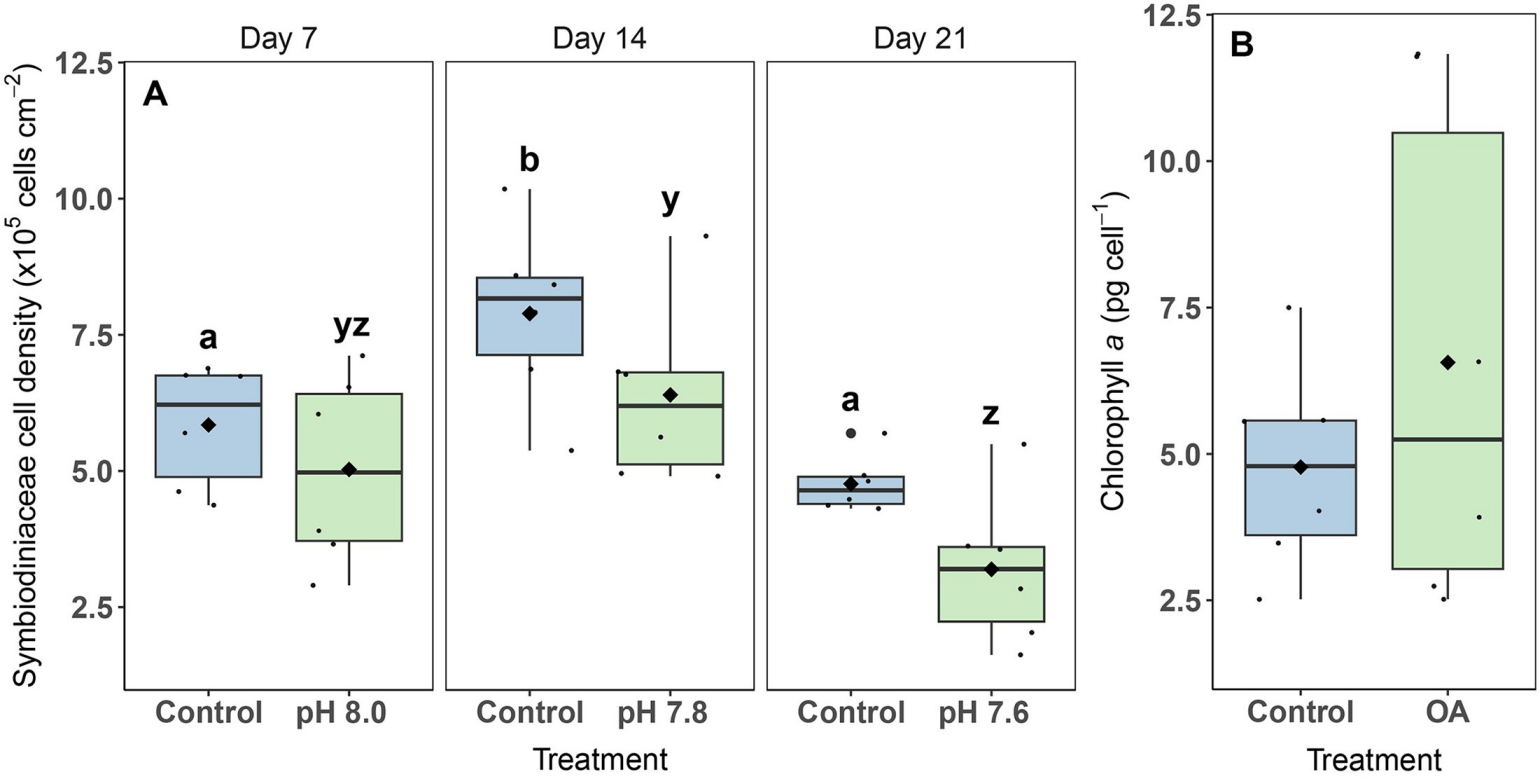
Symbiodiniaceae cell density (A) and chlorophyll *a* content (B) of *Xenia umbellata* exposed to ocean acidification (OA). The black horizontal line in each boxplot represents the median, while the black diamond represents the mean. Blue boxplots are controls; green boxplots are the OA treatments. Small black circles represent data points (n_treatment_ = 6), and big black circles represent outliers. For (A): Significant differences (p < 0.05) within treatments between days are shown by different letters, while no significant differences were found between treatments per day. For (B): No significant differences were found between treatments.

In general, cell densities were always lower in the OA treatment compared to the control, but not significantly (Fig 3A). Significant main effects were found for Treatment (2-way ANOVA, F_2,30_ = 15.1, p < 0.001) and Day (2-way ANOVA, F_1,30_ = 7.6, p = 0.010), but pairwise comparisons were only significant for changes in Symbiodiniaceae cell densities, and not between the control and the OA treatment. On day 7, cell densities for the control and OA treatment were 5.85 ± 0.46 and 5.03 ± 0.72 x10^5^ cells cm^-2^, respectively, which increased on day 14 by 35% and 27%, respectively, and decreased on day 21 by 40% and 50%, respectively, compared to day 14 (Fig 3A).

Chlorophyll *a* content was not significantly different between control (4.77 ± 0.73 pg cell^-1^) and OA exposed colonies (6.56 ± 1.76 pg cell^-1^) at the end of the experiment (t-test, p = 0.371) (Fig 3B).

### Oxygen fluxes

**Fig 4:**
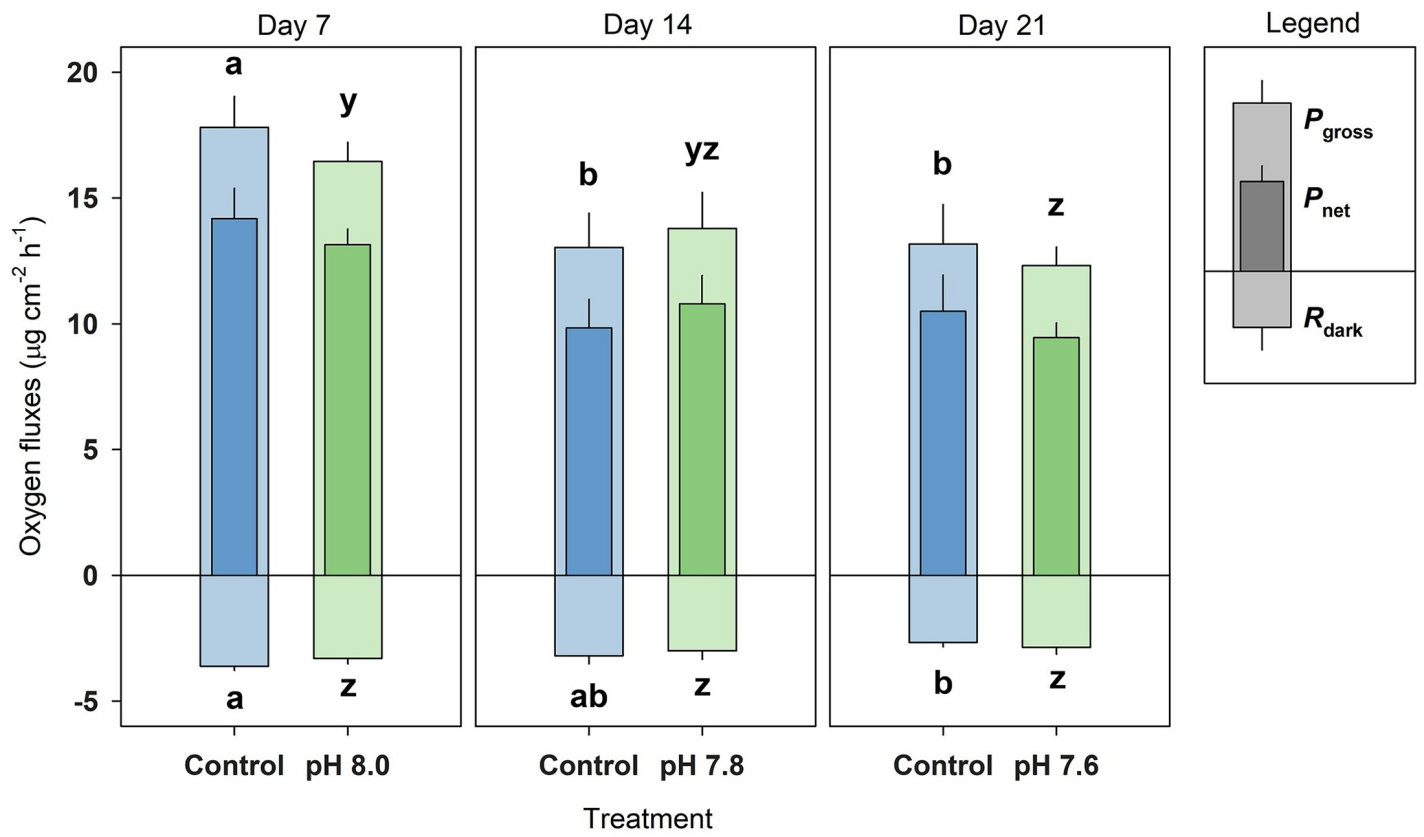
Oxygen fluxes of *Xenia umbellata* exposed to ocean acidification (OA). *P*_gross_ = gross photosynthesis, *P*_net_ = net photosynthesis, and *R*_dark_ = dark respiration. Blue bars represent the control; green bars represent the OA treatments. Bars are mean ± standard error (n_treatment_ = 6). Significant differences (p < 0.05) within treatments between days are shown by different letters (*P*_gross_ and *P*_net_ share the same significance letters), while no significant differences were found between treatments per day.

A significant main effect was found for Day for *P*_net_ (2-way RM ANOVA, F_2,20_ = 8.5, p = 0.002), *P*_gross_ (2-way RM ANOVA, F_2,20_ = 8.8, p = 0.002), and *R*_dark_ (2-way RM ANOVA, F_2,20_ = 5.9, p < 0.009), but not for Treatment nor was there an interaction of Day and Treatment. In general, *P*_net_, *P*_gross_, and *R*_dark_ decreased after Day 7 by ~28%, ~26% and ~19%, respectively, for Day 14 and 21 (Fig 4). No significant difference between control and OA treatment was found for any day.

### Stable isotope signatures and CN content

**Fig 5:**
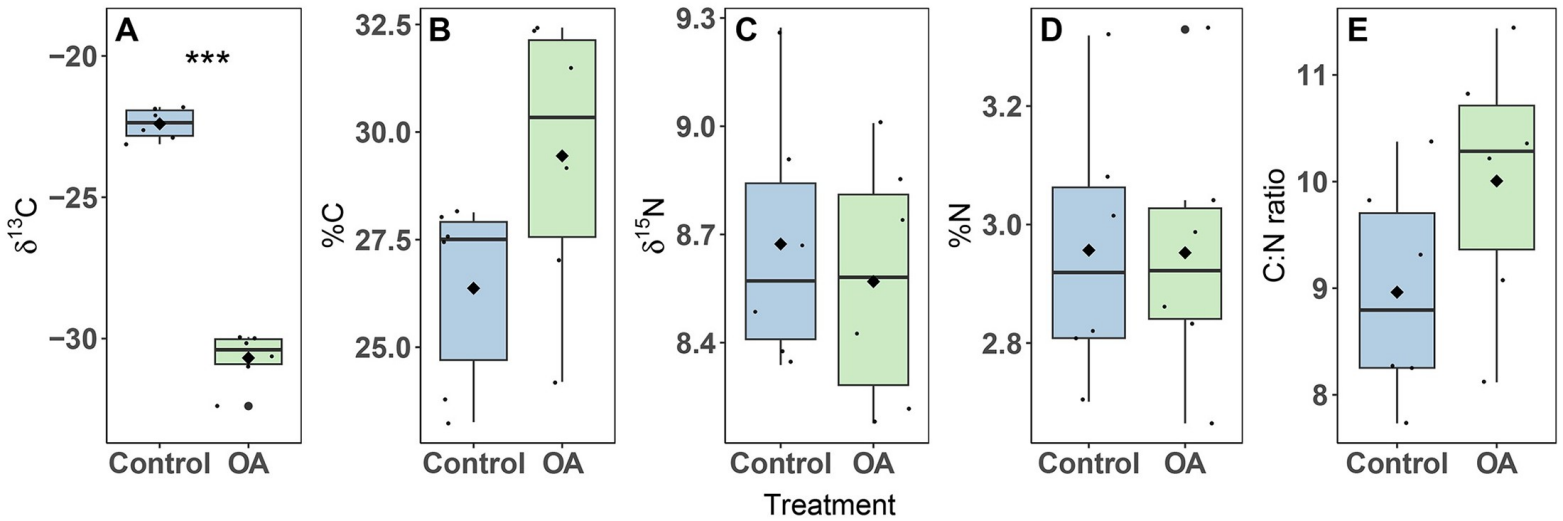
Stable isotope signature of carbon (δ^13^C)(A), elemental composition of carbon (%C)(B), stable isotope signature of nitrogen (δ^15^N)(C), elemental composition of nitrogen (%N)(D) and ratio of carbon and nitrogen (E) of the *Xenia umbellata* holobiont exposed to ocean acidification (OA) at day 21. The black horizontal line in each boxplot represents the median, while the black diamond represents the mean. Blue boxplots are controls; green boxplots are the OA treatments. Small black circles represent data points (n_treatment_ = 6), and big black circles represent outliers. Significant differences between treatments are shown by asterisks (*** p < 0.001).

The δ^13^C of OA exposed colonies significantly decreased compared to the control (t-test, p < 0.001), on average by 8.3 ‰ (Fig 5A). Colonies exposed to OA revealed a non-significant (t-test, p = 0.09) increase in %C compared to controls by ~3% (Fig 5B). Nitrogen isotopes (δ^15^N) remained stable at 8.6 ± 0.1 ‰ and 8.7 ± 0.1 ‰ for the OA and control colonies, respectively (Fig 5C). The percentage of N in the holobiont remained stable at 3.0 ± 0.1 for both treatments (Fig 5D). The C:N ratio was higher in the OA treatment (10.0 ± 0.5), but not significantly (t-test, p = 0.14) compared to the control (9.0 ± 0.4) (Fig 5E).

## Discussion

Previous studies reported marginal effects of OA on soft coral health (Table 2), while none of these studies (except for one observation) reported altered physiology of xeniids in response to OA. In the present study, we found reduced pulsation and growth of the xeniid *X*. *umbellata* in response to OA. We posit that pulsation may be a very beneficial and phenotypically plastic trait for xeniids when exposed to higher *p*CO_2_ concentrations. As a result, pulsating xeniids may become even more dominant under future climate change scenarios.

**Table 2:** Comparison of results of previous ocean acidification experiments/observations using soft corals.

Soft coral species	Family	pH exposures	Exposure length	Affected response variables	Non-affected response variables	Reference
*Xenia umbellata* ^p^	Xeniidae	8.3, 8.0, 7.8, 7.6	7 days per OA treatment	Reduced pulsation rate, growth rate and δ^13^C	Visual coloration, survival, Symbiodiniaceae cell density, chl. *a* content, *P*_net_, *P*_gross_, *R*_dark_, δ^15^N, %C, %N, C:N content	Present study
*Xenia* sp.^p^	Xeniidae	< 8.1	Longer periods	Uncoordinated pulsation*	N/A	[62]
*Corallium rubrum*	Coralliidae	8.09, 7.88, 7.77	10 and 45 days	Reduced biocalcification, growth rates and feeding	-	[63]
*Corallium rubrum*	Coralliidae	8.1, 7.81	314 days	Spicule morphology, reduced growth rate	Carbohydrate, lipid, protein and fatty acid composition	[64]
*Ovabunda macrospiculata* ^p^	Xeniidae	8.2, 7.6, 7.3	30–90 days	-	Symbiodiniaceae cell density and chl. *a* content, sclerite weight;polyp weight, pulsation rate, polyp weight	[26]
*Heteroxenia fuscescens* ^p^	Xeniidae	8.2, 7.6, 7.3	30–90 days	-	Symbiodiniaceae cell density and chl. *a* content	
*Sarcophyton* sp.	Alcyoniidae	8.2, 7.6, 7.3	~150 days	-	Symbiodiniaceae cell density and chl. *a* content	
*Ovabunda macrospiculata* ^p^	Xeniidae	8.2, 7.6, 7.3	42 days	-	Sclerite microstructure	[27]
*Eunicea fusca*	Plexautidae	8.1–7.1	28 days	Growth and calcification both decreased with decreasing pH	-	[65]
*Sarcophyton glaucum*	Alcyoniidae	8.2, 8.0, 7.8	3 days	Reduced cytotoxic compounds (only at pH 7.8)	Cytotoxic compounds (at pH 8.0)	[28]
*Veretillum cynomorium*	Veretillidae	8.0, 7.7	60 days	-	Antioxidant enzymes, lipid peroxidation, heat shock response	[66]
*Rhytisma fulvum*	Alcyoniidae	~8.1, ~7.9, ~7.7	49 days	Reduced maximum relative electron transport rate	Alpha, Fv/Fm, Ek, NPQmax	[29]

N = Nitrogen

C = Carbon

*P*_net_ = net photosynthesis

*P*_gross_ = gross photosynthesis

*R*_dark_ = dark respiration

Chl. *a* = Chlorophyll *a*

^p^ = Pulsating xeniids

*Observation made by the book authors, not part of an empirical study

### Acidification did not affect the photophysiology of *X*. *umbellata*

The δ^13^C of the OA exposed colonies revealed a significant decrease compared to the control colonies, by ~8.3 ‰, indicating a higher uptake and incorporation of the lighter ^12^C isotope from atmospheric CO_2_ into the holobiont [55]. Lighter C isotope signatures were also found in hard corals exposed to OA [42], thus indicating similar responses between hard and soft corals. However, this increased incorporation, and ultimately availability, of DIC was not reflected in the C content or the C:N ratios, highlighting that C was likely not the limiting nutrient for coral productivity [43]. The higher availability and a subsequent alleviation of C limitation would have theoretically resulted in higher numbers of Symbiodiniaceae cells through the use of before unused N [56, 57]. Though, in the present study, cell densities of Symbiodiniaceae, as well as chl. *a* content of Symbiodiniaceae, in OA exposed colonies were not significantly different from the controls. Concentrations of environmental dissolved inorganic N also remained stable (Table 1). We can thus conclude that N was likely the limiting nutrient for primary production throughout the entire experiment. This was further evidenced by stable *P*_net_, *P*_gross_ and *R*_dark_ between control and OA treatment. As pulsation and the fluxes of oxygen may be linked [37], we expected pulsation rates to remain stable as well [26].

### Acidification affected pulsation and growth of *X*. *umbellata*

Even though the photophysiology of the holobiont remained unaffected, pulsation was significantly affected. Pulsation rates gradually decreased with every decrease in water pH compared to the control at the same stage, i.e., a decrease of 17% at pH 8.0, 26% at pH 7.8 and 32% at pH 7.6. Previous studies have reported reductions of *Xenia* spp. pulsation rates in response to warming [39], heavy nitrate eutrophication [38], a lack of a heterotrophic food source [41], and exposure to oil dispersants [40]. The synchronous opening and contracting of the polyp tentacles, in a continuous rhythm, results in a water flow that enhances photosynthesis by rapidly removing excess oxygen while increasing CO_2_ affinity of ribulose-1,5-bisphosphate carboxylase oxygenase (RuBisCO) and preventing refiltration of surrounding water by neighboring polyps [36, 37, 58]. As such, a decrease in pulsation rates should theoretically have resulted in decreased photosynthesis, which remained unaffected. We therefore hypothesize that *X*. *umbellata* reduced its pulsation to compensate for the higher availability of DIC [59], thus reducing gas exchange to maintain stable productivity, which may have ultimately reduced the effects of OA on the corals’ photophysiology.

As pulsation is an energy costly process [36], the reduction in pulsation rates could preserve energy for other vital processes such as growth. However, the observed reduction in pulsation rates associated with OA exposed colonies was accompanied with a decrease in SGR compared to controls. Growth rates in OA treatments decreased by 58% and 65%, respectively, during the second and third week compared to the first week of the experiment. Although growth rates in control tanks were also reduced in the second and third week (by 57% and 53%, respectively), this was less pronounced than the decline in OA treatments. We speculate that both treatments experienced an unknown factor affecting both treatments, e.g., a lower availability of organic and/or inorganic nutrients, not measurable by our analytic tests, that are available in the holding tank of the mother colonies [53]. This lower availability of nutrients may have been exacerbated by diminished pulsation rates associated with OA exposed colonies, though only at pH 7.6 in the third week. Indeed, lower pulsation rates and subsequent increased water refiltration by adjacent polyps may have reduced the uptake of particulate (e.g., detritus, small phyto- and zooplankton) and dissolved (e.g., small carbohydrates, amino and fatty acids) organic matter, as well as the supply of inorganic N and phosphorus (P), which are essential for soft coral growth [37]. In addition, the energy obtained from translocated photosynthates by the Symbiodiniaceae may have been redirected to other processes, e.g., mucus production, in the holobiont rather than invested in growth. Translocation of photosynthates may even increase under OA as found for the hard coral *Stylophora pistillata*, though this was accompanied by reduced cell densities of the Symbiodiniaceae and their chl. *a* content [60]. This is less likely to have happened in the present study as both remained stable. The opposite, i.e., a reduction in photosynthate translocation, would theoretically be possible, but could not be inferred with the reported response variables in our study. Future studies could shed light on resource acquiring/partitioning by separating the coral tissue and Symbiodiniaceae for isotope and elemental analyses, use labelled isotopes of C and N, and/or by performing nutrient uptake incubations. Taken together, our results suggest that *X*. *umbellata* had less nutrients and/or energy available for growth under OA conditions, which could have been induced by lower pulsation rates and/or altered use of translocated photosynthates.

### Comparison to previous studies on octocorals

Previous studies have shown that xeniids could be protected from OA as their tissue may act as a protective barrier [26, 27]. Our results suggest that this is not necessarily the case for *X*. *umbellata* since pulsation- and growth rates in the present study were affected, thus partially contrasting Gabay and colleagues [26] (Table 2). In their study, while exposing the pulsating xeniid *Ovabunda macrospiculata* to pH values of 7.6 and 7.3, pulsation and growth both persisted (though caution is needed as they quantified growth as “sclerite weight to polyp weight”). Hence, different species can show different responses to stressors, even if they belong to the same family and may therefore be more similar in physiology [61]. To our knowledge, only one observation mentions flaccid and unhealthy-looking *Xenia* sp. with less coordinated pulsation in response to lower pH exposure (8.1 and lower) [62]. In the present study, colonies remained visually healthy and continued to pulsate in a coordinated manner. In accordance with Gabay and colleagues [26], Symbiodiniaceae cell density and chl. *a* content remained unaffected, while another study on hard corals found reductions in both response variables [60].

### Ecological implications

In the present study, we found no effect of strong OA (pH 7.6) on the photophysiology of *X*. *umbellata*, while pulsation and growth rates were significantly impacted compared to controls. Octocorals that have been exposed to longer periods of ecologically relevant pH values (i.e., above ~7.8) have shown little to no negative effects (Table 2). In contrast, though in accordance with the present study, hard corals have shown reductions of 50% in skeletal growth at similar pH ranges [10, 67]. Previous research on octocorals (Table 2) suggests that the calcium carbonate microstructures (i.e., the sclerites) of octocorals maintain their integrity under OA conditions [26, 27]. However, to better predict the success of *X*. *umbellata* under future OA scenario, future studies should assess the dissolution and production rates of the sclerites as well as their microstructure, i.e., morphology [e.g., 27]. If the sclerites of *X*. *umbellata* also maintain their integrity under OA conditions, a reduction in growth is only a small price to pay to remain viable. Ocean acidification is a gradual process where pH will decrease mostly linearly over the next decades rather than abruptly within days. Therefore, the results obtained in the present short-term study may differ from long-term effects on *X*. *umbellata*. However, soft corals [68, 69], including xeniids [70], can inhabit waters surrounding CO_2_ seeps, thus providing evidence for their potential long-term viability under higher *p*CO_2_. Differences in light intensities due to large depth ranges of xeniids [71], could potentially interact with acidification and further influence the physiological responses. Overall, caution is required in extrapolating data of the present study to imply natural ecological effects pertaining to long-term OA. We thus recommend long-term OA studies with *X*. *umbellata* combined with different light intensities to accurately assess the response of this resilient soft coral species to expected future conditions. However, our results do suggest that *X*. *umbellata* will remain viable during short-term naturally occurring acidification events, e.g., large diel fluctuations in pH [72], or low water pH exposure from (seasonal) upwelling events [73].

*Xenia umbellata* has been subject to experiments assessing the (combined) effects of global and/or local factors; e.g., dissolved organic C eutrophication and warming [39, 74, 75], nitrate eutrophication and warming [38], phosphate eutrophication and warming [53, 76], water flow and food availability [41], and *in-situ* eutrophication [54, 77]. Future scenarios for corals will likely include multiple global and local factors, with ocean warming as the most urgent threat. In some of the previously mentioned studies, the addition of a local factor mitigated the effects of ocean warming [39, 53, 76], while others exacerbated the effects of ocean warming [38]. Future studies should also investigate combined effects of multiple global factors that are expected to happen simultaneously, i.e., ocean warming and acidification [78]. When there are too many stress factors, shifts in community composition may happen, where for example, soft corals will replace hard corals [79]. Xeniids in particular are rapid, opportunistic colonizers of disturbed habitats, especially coral relicts [34], which are relatively resistant to ocean warming [38, 39]. Their rapid clonal growth through a strategy of larval incubation and effective asexual reproduction, as well as the production of allelopathic substances that chemically inhibits the growth of other organisms, helps them spread widely [32–34]. This shift away from hard corals can have harmful effects because they provide complex three-dimensional habitats for other organisms [9, 69]. Therefore, a wide distribution of soft corals has consequences for the functioning of the whole reef.

In conclusion, we posit that *X*. *umbellata* may adjust to acidified water by altering its pulsation activity, highlighting the phenotypic plasticity of this trait. We further posit that *X*. *umbellata* will remain viable during short-term pH fluctuations, whilst cautiously interpreting the results of the present study to *X*. *umbellata*’s success under long-term OA conditions. However, based on results obtained here and elsewhere [26, 69, Table 2], we hypothesize that *X*. *umbellata* will have a competitive advantage over hard corals under future climate change scenarios, though long-term studies are required to confirm this.

## Supporting information

S1 Fig Overview of the acidification setup. A total of two of these setups acidified the water of six tanks.
(DOCX)

S1 Data (XLSX)
