# Supplementary material for: Short-term ocean acidification decreases pulsation and growth of the widespread soft coral Xenia umbellata
Source: PLoS One. 2023 Nov 15;18(11):e0294470. doi: 10.1371/journal.pone.0294470 (PMC10651030; doi:10.1371/journal.pone.0294470)
Supplement: S1 Fig — A total of two of these setups acidified the water of six tanks. (DOCX) [file pone.0294470.s001.docx]

**Supporting information**

**Supplementary Figures**

**
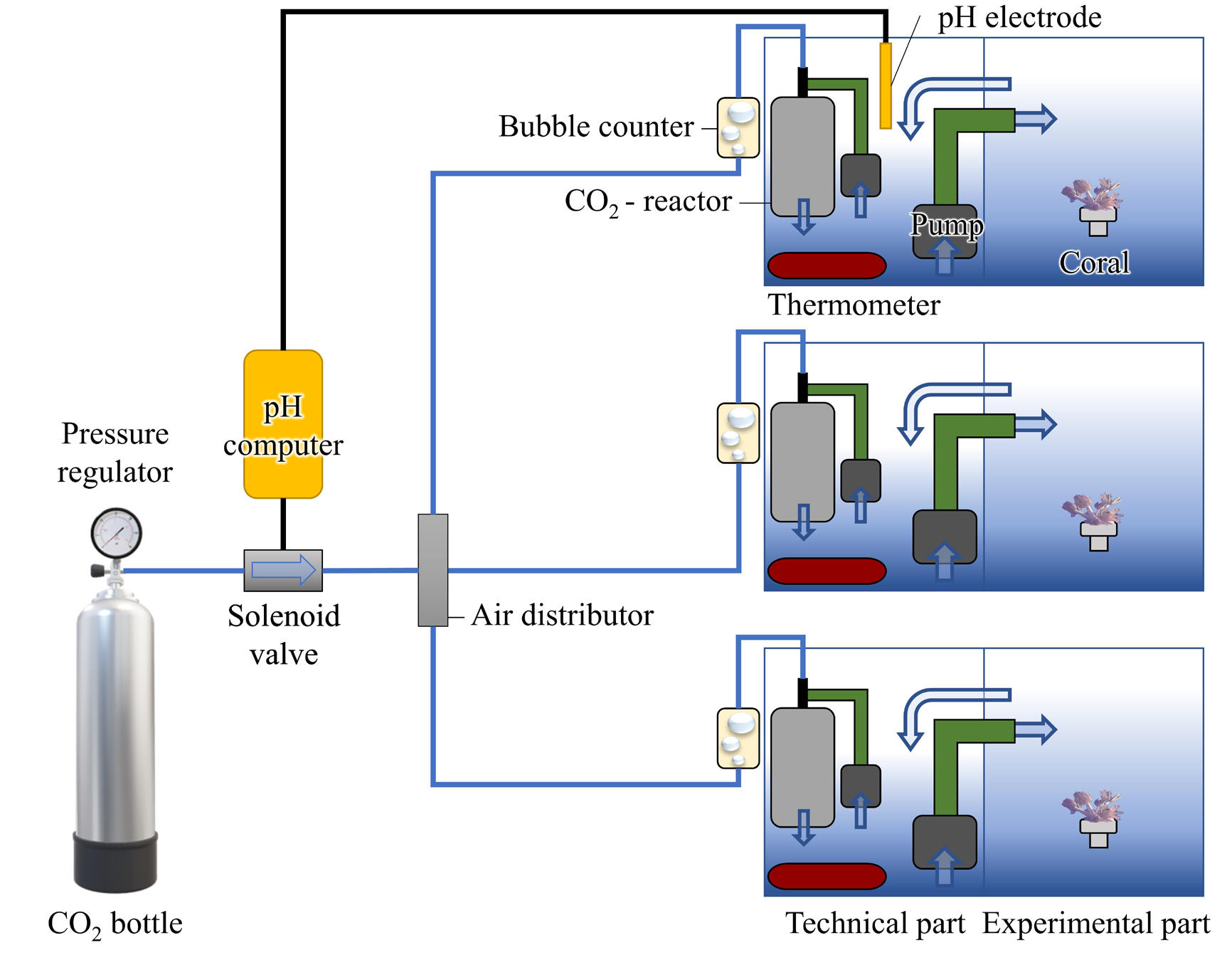
**

**S1 Fig. Overview of the acidification setup.** A total of two of these setups acidified the water of six tanks.
